# Gender differences in waterpipe tobacco smoking among university students in four Eastern Mediterranean countries

**DOI:** 10.18332/tid/129266

**Published:** 2020-12-02

**Authors:** Randah R. Hamadeh, Juhan Lee, Niveen M. E. Abu-Rmeileh, Muhammad Darawad, Aya Mostafa, Khalid A. Kheirallah, Afzalhussein Yusufali, Justin Thomas, Mohamed Salama, Rima Nakkash, Ramzi G. Salloum

**Affiliations:** 1 College of Medicine and Medical Sciences, Arabian Gulf University, Manama, Bahrain; 2 Department of Health Education and Behavior, College of Health and Human Performance, University of Florida, Gainesville, United States; 3 Institute of Community and Public Health, Birzeit University, Birzeit, Occupied Palestinian Territories; 4 School of Nursing, The University of Jordan, Amman, Jordan; 5 Department of Community, Environmental and Occupational Medicine, Ain Shams University, Cairo, Egypt; 6 Department of Public Health, Jordan University of Science and Technology, Irbid, Jordan; 7 Hatta Hospital, Dubai Medical College, Dubai, United Arab Emirates; 8 Department of Psychology, Zayed University, Abu Dhabi, United Arab Emirates; 9 Institute of Global Health and Human Ecology, The American University in Cairo, New Cairo, Egypt; 10 Faculty of Health Sciences, American University of Beirut, Beirut, Lebanon; 11 Department of Health Outcomes and Biomedical Informatics, College of Medicine, University of Florida, Gainesville, United States

**Keywords:** waterpipe, shisha, hookah, youth, EMR

## Abstract

**INTRODUCTION:**

Males have a higher prevalence of waterpipe tobacco smoking (WTS) than females in most Eastern Mediterranean Region (EMR) countries, with a smaller gender gap than that of cigarette smoking. The objective of this study was to determine gender differences among university students with respect to WTS initiation, smoking behavior, tobacco flavors, and expenditure on WTS, in four EMR countries.

**METHODS:**

A cross-sectional online survey was conducted based on convenient samples of ever waterpipe smokers among university students in four EMR countries (Egypt, Jordan, Occupied Palestinian Territories, and the United Arab Emirates) in 2016. The total samples included 2470 participants. Study participants were invited through flyers, university portals, emails and Facebook, followed by emails with links to the internet survey.

**RESULTS:**

Females (80.4%) were more likely than males (66.4%, p<0.001) to be in the younger age group (18–22 years) and they were less likely to be current waterpipe smokers (females, 60.0%; males 69.5%, p<0.001). Two-thirds of students across both genders smoked their first waterpipe at the age of 15–19 years, with more females starting with family members. Over one-third of males and 14.9% of the females usually smoked ≥10 heads (p<0.001). About half (46.6%) of females smoked for less than half an hour compared to 30.5% of males (p<0.001). Only 1% of females smoked non-flavored tobacco compared to 11% of males (p<0.001). There was a significant (p=0.05) positive correlation (r=0.808) with respect to tobacco flavor usually smoked between males and females with apple/double apple being the most popular.

**CONCLUSIONS:**

There were gender differences in WTS in several aspects. The study has implications for educational establishments, tobacco control and women civil society groups, as well as policymakers.

## INTRODUCTION

The prevalence of waterpipe tobacco smoking (WTS) in the Eastern Mediterranean Region (EMR) is on the rise especially among youth^1^. Of the 25 countries that assessed WTS in the Global Youth Tobacco Survey among students aged 13–15 years, Lebanon (36.9%) and the West Bank (32.7%) had the highest prevalence rates^1^. Although WTS prevalence is higher in males in most EMR countries, the gender gap is much smaller than that of cigarette smoking^2,3^. In a number of countries in the EMR, restrictions towards smoking are religious, cultural and social, and are more firmly applied to females than males, and to cigarette smoking compared with WTS^4^. Recent evidence from Egypt indicates that the gender gap for certain tobacco product types is narrowing and even higher among females for electronic nicotine delivery systems (ENDS) use^5^. Previous studies have attributed this finding partially to the social acceptability of non-cigarette tobacco products compared with cigarettes^6-8^. Further, the perception of reduced harm and the availability of flavored waterpipe tobacco are among the reasons for its social acceptability^8^. It is common for women in the EMR to report WTS initiation within their families^8,9^. In Turkey, the presence of waterpipe smokers among family members was found to have significant effects on the prevalence rate of WTS amongst university students^10^.

Flavored waterpipe tobacco (‘maassel’) is the most preferred tobacco by waterpipe smokers globally, particularly among women^11,12^. In young people, flavors are reported as a common reason to experiment with WTS^9^. The World Health Organization (WHO) warned that women and girls are a major target for the tobacco industry to recruit new users and the rise in smoking among women has been associated with industry promotion of flavored tobacco^13^.

Given that there have not been sufficient studies comparing the behaviors of men and women in the EMR with respect to WTS, the objective of this study was to determine if there were gender differences among university students with respect to WTS initiation, smoking behavior, waterpipe tobacco flavors, and expenditure on WTS, in four EMR countries.

## METHODS

A cross-sectional online survey was conducted based on convenience samples of ever waterpipe smokers among university students in four EMR countries: Egypt (2 universities), Jordan (2 universities), Occupied Palestinian Territories (OPT, 1 university), and the United Arab Emirates (UAE, 2 universities), between April and December 2016. The two universities from Egypt had an enrolment of 91041 and 168970, those of Jordan 20000 and 37692, and those of the UAE 500 and 9217, and 13963 for the OPT^14^. An estimated sample size of 384 students from each country was computed based on a 50% prevalence of WTS and a 95% confidence interval. We targeted a sample of 400 participants per site to ensure adequate power for another component of this survey, reported elsewhere^14,15^.

We recruited student samples that were heterogeneous with respect to gender and study level (undergraduate vs graduate). Participants were initially invited through flyers, university portals, emails and Facebook. Students then received emails with links to the internet survey available in both Arabic and English and they were assured that their responses would remain anonymous. Participants were further assured that their smoking status would not be revealed and that they could leave the survey at any time. Financial incentives were not provided for participation in the survey. The study protocol was approved by the institutional review boards of all participating institutions. All study participants had to consent before taking the survey. The original dataset used and analyzed during the current study is available from an author (RGS), on reasonable request.

The inclusion criteria were age (≥18 years), currently enrolled in the university or will be in the next year, and had ever smoked waterpipe for at least one or two puffs. More details on the methods of student recruitment at the participating universities are mentioned elsewhere^14-16^. Students who were not eligible or did not complete the online consent form were excluded.

The questionnaire was based on a standard survey on WTS and included demographic characteristics of waterpipe smokers, WTS history, WTS initiation, current use of waterpipe including flavors, in addition to concurrent use of other tobacco products. Current waterpipe smokers and current cigarette smokers were defined as those who smoked in the last 30 days prior to the survey. Students who smoked the waterpipe the day of the survey or the previous day were considered daily smokers.

We compared males and females in all countries by: sociodemographic characteristics; tobacco use status within last 30 days by type; waterpipe smoking initiation by age, person(s) and place; most recent WTS session by duration of smoking; number of waterpipe heads, and place smoked; usual persons smoking waterpipe with, and places of smoking and purchase; and the top five waterpipe flavors usually smoked. Means and standard deviations were calculated for continuous variables and independent sample t-test was applied. SPSS version 22 was used for conducting the statistical analysis. Chi-squared test was used to determine gender differences within each country and overall, for the categorical variables. Spearman correlation (r) was calculated for the ranks of waterpipe flavors between genders. Differences were considered significant for p-value <0.05.

## RESULTS

The number of the university students that participated in the study was 2470 across the 4 countries. There were 728 respondents from Egypt (27.5% females), 790 from Jordan (37.1% females), 772 from the OPT (52.1% females), and 180 from the UAE (42.8% females).

### Demographic characteristics

There were statistically significant differences between females and males with respect to age, educational level, marital status and employment, in the total sample (Table 1). More female students (80.4%) were in the younger age group (18–22 years) than male students (66.4%) (p<0.001). Bachelor’s, non-medical degree, was the highest level attained by female (84.0%) and male (72.3%) students (p<0.001) with equal percentages in the OPT but higher among females in the other countries. More females were unmarried than males with the sample from Egypt having the lowest proportions of unmarried across both genders. The gender gap in employment status was high in Jordan, the OPT, and in the overall sample (p<0.001), with more males being employed than females. Egypt had the highest employment among its female and male students. The majority of male (92.7%) and female (93.9%) students were citizens of their respective countries of residence.

**Table 1:** Demographic characteristics of study participants in four EMR countries, 2016 (N=2470)

*Characteristics*	*Jordan*		*OPT*		*UAE*		*Egypt*		*Total*	
	*Female n (%)*	*Male n (%)*	*Female n (%)*	*Male n (%)*	*Female n (%)*	*Male n (%)*	*Female n (%)*	*Male n (%)*	*Female n (%)*	*Male n (%)*
**Age** (years)										
18–22	229 (81.2)	393 (80.7)	346 (88.9)	303 (83.0)	60 (78.9)	82 (83.7)	20 (29.4)	203 (38.4)	655 (80.4)	981 (66.4)
≥23	53 (18.8)	94 (19.3)	43 (11.1)	62 (17.0)	16 (21.1)	16 (16.3)	48 (70.6)	325 (61.6)	160 (19.6)	497 (33.6)
Total	282	487	389	365	76	98	68	528	815	1478
p-value*	0.863		0.019		0.425		0.147		<0.001	
**Degree level**										
Diploma	17 (5.9)	73 (14.9)	10 (2.6)	9 (2.5)	4 (5.2)	16 (15.8)	3 (4.9)	59 (12.1)	34 (4.2)	157 (11.0)
Bachelor’s, non-medical	236 (81.7)	323 (66.1)	340 (89.5)	316 (89.3)	60 (77.9)	73 (72.3)	42 (68.9)	324 (66.4)	678 (84.0)	1036 (72.3)
Medical	29 (10.0)	78 (16.0)	1 (0.3)	3 (0.8)	9 (11.7)	7 (6.9)	5 (8.2)	32 (6.6)	44 (5.5)	120 (8.4)
Graduate	7 (2.4)	15 (3.0)	29 (7.6)	26 (7.3)	4 (5.2)	5 (5.0)	11 (18.0)	73 (15.0)	51 (6.3)	119(8.3)
Total	289	489	380	354	77	101	61	488	807	1432
p-value	<0.001		0.759		0.127		0.381		<0.001	
**Marital status**										
Never married	283 (96.9)	479 (96.6)	364 (93.3)	355 (96.7)	74 (96.1)	97 (95.1)	57 (83.8)	403 (77.4)	778 (94.1)	1334 (89.8)
Ever married	9 (3.1)	17 (3.4)	26 (6.7)	12 (3.3)	3 (3.9)	5 (4.9)	11 (16.2)	118 (22.6)	49(5.9)	152(10.2)
Total	292	496	390	367	77	102	68	521	827	1486
p-value	0.793		0.032		0.747		0.225		<0.001	
**Employment status**										
Unemployed	225 (82.1)	315 (68.8)	271 (78.1)	196 (59.6)	60 (78.9)	71 (71.7)	24 (41.4)	200 (41.9)	580 (76.8)	782 (57.4)
Employed	49 (17.9)	143 (31.2)	76 (21.9)	133 (40.4)	16 (21.1)	28 (28.3)	34 (58.6)	277 (58.1)	175(23.2)	581(42.6)
Total	274	458	347	329	76	99	58	477	755	1363
p-value	<0.001		<0.001		0.274		0.936		<0.001	
**Citizenship status**										
Citizen	267 (91.8)	434 (88.0)	387 (99.5)	366 (99.2)	53 (68.8)	58 (56.9)	68 (100.0)	522 (99.4)	775 (93.9)	1380 (92.7)
Non-citizen/expatriate	24 (8.2)	59 12.0)	2 (0.5)	3 (0.8)	24 (31.2)	44 (43.1)	0 (0.0)	3 (0.6)	50 (6.1)	109 (7.3)
Total	291	493	389	369	77	102	68	525	825	1489
p-value	0.102		0.611		0.102		0.532		0.251	

OPT: Occupied Palestinian Territories. UAE: United Arab Emirates. The numbers vary by each variable due to missing values.

* p-values for all characteristics were derived from chi-squared tests.

### Current waterpipe and other types of tobacco smoking

There were more current smokers of any type of tobacco among males (84.9%) than females (66.2%) in the overall sample (p<0.001) and within each country, with the smallest gender gap in Egypt.

The UAE had the lowest proportion of current waterpipe smokers among females (49.4%) and males (51.5%). Egypt had the largest gender gap in current WTS (males 58.7%; females 77.2%).

All female waterpipe smokers in Egypt and the UAE were non-daily smokers (Table 2). Electronic waterpipe smoking among males (11.9%) was almost double that of their female counterparts (6.6%) with the highest difference observed in the UAE.

**Table 2:** Tobacco use status (within last 30 days) by type in four EMR countries, 2016 (N=2470)

*Tobacco use*	*Jordan*		*OPT*		*UAE*		*Egypt*		*Total*	
	*Female n (%)*	*Male n (%)*	*Female n (%)*	*Male n (%)*	*Female n (%)*	*Male n (%)*	*Female n (%)*	*Male n (%)*	*Female n (%)*	*Male n (%)*
**Any tobacco**										
Current user	203 (70.5)	412 (84.1)	244 (63.0)	301 (82.0)	40 (51.9)	81 (81.0)	54 (83.1)	451 (88.6)	541 (66.2)	1245 (84.9)
Former user	85 (29.5)	78 (15.9)	143 (37.0)	66 (18.0)	37 (48.1)	19 (19.0)	11 (16.9)	58 (11.4)	276 (33.8)	221 (15.1)
Total	288	490	387	367	77	100	65	509	817	1466
p-value*	<0.001		<0.001		<0.001		0.197		<0.001	
**Waterpipe**										
Current, non-daily smoker	171 (59.8)	279 (58.2)	215 (55.7)	200 (55.2)	38 (49.4)	51 (51.5)	37 (58.7)	368 (74.8)	461 (56.8)	898 (62.7)
Current, daily smoker	14 (4.9)	54 (11.3)	12 (3.1)	43 (11.9)	0 (0.0)	2 (2.0)	0 (0.0)	12 (2.4)	26 (3.2)	111 (7.8)
Former smoker	101 (35.3)	146 (30.5)	159 (41.2)	119 (32.9)	39 (50.6)	46 (46.5)	26 (41.3)	112 (22.8)	325 (40.0)	423 (29.5)
Total	286	479	386	362	77	99	63	492	812	1432
p-value	0.008		<0.001		0.416		0.004		<0.001	
**Electronic waterpipe (e-hookah)**										
Current user	28 (9.9)	52 (11.0)	13 (3.4)	7 (2.0)	3 (3.9)	18 (18.2)	9 (14.8)	89 (18.9)	53 (6.6)	166 (11.9)
Former user	25 (8.8)	58 (12.3)	7 (1.8)	10 (2.8)	21 (27.6)	16 (16.2)	10 (16.4)	45 (9.5)	63 (7.9)	129 (9.2)
Never user	231 (81.3)	363 (76.7)	360 (94.7)	335 (95.2)	52 (68.4)	65 (65.7)	42 (68.9)	338 (71.6)	685 (85.5)	1101 (78.9)
Total	284	473	380	352	76	99	61	472	801	1396
p-value	0.268		0.339		0.007		0.221		<0.001	
**Cigarettes**										
Current smoker	55 (19.2)	230 (47.3)	57 (15.1)	168 (46.7)	16 (21.1)	55 (55.0)	27 (47.4)	263 (55.1)	155 (19.4)	716 (50.3)
Former smoker	27 (9.4)	40 (8.2)	25 (6.6)	12 (3.3)	15 (19.7)	9 (9.0)	0 (0.0)	20 (4.2)	67 (8.4)	81 (5.7)
Never smoker	204 (71.3)	216 (44.4)	296 (78.3)	180 (50.0)	45 (59.2)	36 (36.0)	30 (52.6)	194 (40.7)	575 (72.1)	626 (44.0)
Total	286	486	378	360	76	100	57	477	797	1423
p-value	<0.001		<0.001		<0.001		0.096		<0.001	
**Electronic cigarettes (e-cigarettes / vape pens)**										
Current user	17 (6.0)	60 (12.5)	2 (0.5)	13 (3.7)	2 (2.6)	18 (18.2)	11 (18.6)	63 (13.5)	32 (4.0)	154 (11.0)
Former user	24 (8.4)	62 (12.9)	15 (4.0)	17 (4.8)	20 (26.0)	18 (18.2)	2 (3.4)	42 (9.0)	61 (7.6)	139 (9.9)
Never user	244 (85.6)	359 (74.6)	360 (95.5)	321 (91.5)	55 (71.4)	63 (63.6)	46 (78.0)	362 (77.5)	705 (88.3)	1105 (79.0)
Total	285	481	377	351	77	99	59	467	798	1398
p-value	0.001		0.009		0.004		0.228		<0.001	
**Cigar**										
Current smoker	21 (7.4)	69 (14.5)	4 (1.1)	38 (10.7)	2 (2.6)	12 (12.1)	9 (15.8)	54 (11.6)	36 (4.5)	173 (12.4)
Former smoker	18 (6.4)	64 (13.4)	16 (4.2)	41 (11.6)	6 (7.9)	18 (18.2)	5 (8.8)	35 (7.5)	45 (5.7)	158 (11.3)
Never smoker	244 (86.2)	343 (72.1)	359 (94.7)	275 (77.7)	68 (89.5)	69 (69.7)	43 (75.4)	376 (80.9)	714 (89.8)	1063 (76.3)
Total	283	476	379	354	76	99	57	465	795	1394
p-value	<0.001		<0.001		0.006		0.600		<0.001	
**Regular pipe**										
Current smoker	24 (8.5)	36 (7.5)	0 (0.0)	5 (1.4)	6 (7.9)	10 (10.2)	3 (4.8)	33 (7.1)	33 (4.1)	84 (6.0)
Former smoker	9 (3.2)	37 (7.8)	7 (1.8)	14 (4.0)	12 (15.8)	15 (15.3)	3 (4.8)	22 (4.7)	31 (3.9)	88 (6.3)
Never smoker	250 (88.3)	404 (84.7)	372 (98.2)	333 (94.6)	58 (76.3)	73 (74.5)	56 (90.3)	413 (88.2)	736 (92.0)	1223 (87.7)
Total	283	477	379	352	76	98	62	468	800	1395
p-value	0.037		0.014		0.872		0.809		0.007	
**Midwakh**										
Current smoker	20 (7.1)	65 (13.7)	0 (0.0)	8 (2.3)	10 (13.2)	52 (52.0)	4 (6.6)	34 (7.2)	34 (4.3)	159 (11.4)
Former smoker	9 (3.2)	46 (9.7)	1 (0.3)	8 (2.3)	12 (15.8)	6 (6.0)	2 (3.3)	24 (5.1)	24 (3.0)	84 (6.0)
Never smoker	254 (89.8)	363 (76.6)	377 (99.7)	334 (95.4)	54 (71.1)	42 (42.0)	55 (90.2)	416 (87.8)	740 (92.7)	1155 (82.6)
Total	283	474	378	350	76	100	61	474	798	1398
p-value	<0.001		0.001		<0.001		0.811		<0.001	
**Smokeless tobacco**										
Current user	21 (7.4)	28 (5.9)	2 (0.5)	4 (1.1)	1 (1.3)	9 (9.4)	3 (5.4)	31 (6.7)	27 (3.4)	72 (5.2)
Former user	4 (1.4)	24 (5.1)	3 (0.8)	5 (1.4)	2 (2.6)	7 (7.3)	4 (7.1)	22 (4.8)	13 (1.6)	58 (4.2)
Never user	259 (91.2)	422 (89.0)	376 (98.7)	343 (97.4)	73 (96.1)	80 (83.3)	49 (87.5)	407 (88.5)	757 (95.0)	1252 (90.6)
Total	284	474	381	352	76	96	56	460	797	1382
p-value	0.028		0.464		0.026		0.704		0.001	

OPT: Occupied Palestinian Territories. UAE: United Arab Emirates. The numbers vary by each variable due to missing values.

* p-values for all types of tobacco use were derived from chi-squared tests.

Current cigarette smoking in males (50.3%) was double that of females (19.4%) in the total sample (p<0.001) and in all countries, with the exception of Egypt. Female students from Egypt had the highest prevalence of cigarette smoking and the smallest gender gap (7.7%) in comparison to Jordan, the OPT, and the UAE. Overall, the prevalence of electronic cigarette use was low among students and more prevalent among males than females, except for Egypt where the prevalence among females was higher than among males and almost equal to that of male students in the UAE (Table 2).

Overall, 10.2% of the females ever smoked cigars compared to 23.7% of males (p<0.001). Lower proportions were noted for regular pipe smoking (8% and 12.3%, respectively). Of the female students, 7.3% ever smoked midwakh compared to 17.4% of the males (p<0.001). UAE males had the highest prevalence of midwakh ever smokers (58%) and current smokers (52%) as well as the UAE females (28.9% and 13.2%, respectively). Smokeless tobacco was not commonly used by both sexes, with the lowest prevalence noted among females and males in the OPT (Table 2).

### First waterpipe smoking

Over two-thirds of males and females smoked their first waterpipe when they were between the ages of 15 and 19 years (Table 3). The OPT had the highest proportion of males (21.0%) who smoked their first waterpipe in the younger age group (10–14 years) and Egypt the highest proportion (18.5%) among females.

**Table 3:** Waterpipe smoking initiation in four EMR countries, 2016 (N=2470)

*Initiation*	*Jordan*		*OPT*		*UAE*		*Egypt*		*Total*	
	*Female n (%)*	*Male n (%)*	*Female n (%)*	*Male n (%)*	*Female n (%)*	*Male n (%)*	*Female n (%)*	*Male n (%)*	*Female n (%)*	*Male n (%)*
**Age at waterpipe smoking initiation** (years)										
10–14	22 (8.0)	53 (11.4)	41 (11.2)	72 (21.0)	8 (10.8)	13 (14.1)	12 (18.5)	51 (9.9)	83 (10.6)	189 (13.4)
15–19	195 (70.7)	365 (78.3)	262 (71.8)	240 (70.0)	50 (67.6)	74 (80.4)	20 (30.8)	270 (52.6)	527 (67.6)	949 (67.1)
≥20	59 (21.4)	48 (10.3)	62 (17.0)	31 (9.0)	16 (21.6)	5 (5.4)	33 (50.8)	192 (37.4)	170 (21.8)	276 (19.5)
Total	276	466	365	343	74	92	65	513	780	1414
p-value*	<0.001		<0.001		0.008		0.003		0.116	
**People with whom smoked first waterpipe**										
No one	16 (5.9)	32 (6.9)	11 (3.0)	24 (6.9)	0 (0.0)	6 (6.4)	10 (15.4)	88 (17.7)	37 (4.8)	150 (10.7)
A friend	79 (28.9)	167 (36.0)	66 (18.1)	150 (43.2)	20 (26.3)	35 (37.2)	31 (47.7)	228 (46.0)	196 (25.2)	580 (41.4)
Several friends	91 (33.3)	214 (46.1)	104 (28.6)	135 (38.9)	36 (47.4)	48 (51.1)	23 (35.4)	176 (35.5)	254 (32.6)	573 (40.9)
Family members	87 (31.9)	51 (11.0)	183 (50.3)	38 (11.0)	20 (26.3)	5 (5.3)	1 (1.5)	4 (0.8)	291 (37.4)	98 (7.0)
Total	273	464	364	347	76	94	65	496	778	1401
p-value	<0.001		<0.001		<0.001		0.904		<0.001	
**Place smoked first waterpipe**										
Café/restaurant/smoke shop	122 (45.0)	259 (57.7)	135 (35.9)	143 (45.1)	53 (71.6)	80 (87.0)	56 (86.2)	426 (84.9)	366 (46.6)	908 (66.8)
Own home	77 (28.4)	71 (15.8)	168 (44.7)	71 (22.4)	12 (16.2)	5 (5.4)	3 (4.6)	15 (3.0)	260 (33.1)	162 (11.9)
Someone else’s home	68 (25.1)	117 (26.1)	68 (18.1)	90 (28.4)	8 (10.8)	7 (7.6)	4 (6.2)	46 (9.2)	148 (18.8)	260 (19.1)
University accommodation	4 (1.5)	2 (0.4)	5 (1.3)	13 (4.1)	1 (1.4)	0 (0.0)	2 (3.1)	15 (3.0)	12 (1.5)	30 (2.2)
Total	271	449	376	317	74	92	65	502	786	1360
p-value	<0.001		<0.001		0.056		0.781		<0.001	

OPT: Occupied Palestinian Territories. UAE: United Arab Emirates. The numbers vary by each variable due to missing values.

* p-values for all ways of initiation were derived from chi-squared tests.

Females used the waterpipe for the first time mostly with family members (37.4%) and friends (32.6%) while males initiated WTS with a friend or several friends (82.3%). In all countries, the proportion of female students smoking their first waterpipe with family members exceeded that of males with the highest in the OPT (50.3%). The majority of males (66.8%) and females (46.6%) smoked their first waterpipe in a café/restaurant with the highest proportion observed in Egypt (84.9% and 86.2%, respectively). Higher proportions of females smoked their first waterpipe at home (33.1%) than males (11.9%) with the highest being among female students in the OPT.

### Usual persons smoked waterpipe with and usual places of purchase

Overall, 14% of females almost always/always smoked the waterpipe with family members compared to 3.3% of males (p<0.001), with the highest among females in Jordan (17.4%) and the OPT (15.2%). High proportions of females (45.0%) and males (38.3%) in Egypt almost always/always smoked alone compared to other countries. In addition, about one-third of females usually smoked waterpipe in a café/restaurant compared to 45% of males. The percentage of females who almost always/always smoked in a café/restaurant was similar to that of males within each country, with students in Egypt having the highest proportions for both females (76.2%) and males (77.0%). There were statistically significant differences (p<0.001) between males and females with respect to the usual place of purchase of waterpipe tobacco, except in Egypt. More females (38.9%) than males (25.8%) did not purchase the waterpipe products they used, with UAE females and males having the highest proportions of not purchasing the products (Supplementary file, Table S1).

Eleven per cent of male students smoked non-flavored tobacco compared to 1% of females (p<0.001). In Egypt, 27.7% of the male students smoked non-flavored tobacco compared to 8.8% of the females (p=0.001). There was a positive strong correlation (r=0.808) between males and females by the top five ranked waterpipe flavors smoked (p<0.0001). The correlation was also strong for Jordan (r=0.911), the OPT (r=0.890) and Egypt (r=0.802) but moderate for the UAE (r=0.468). Table 4 shows the top five waterpipe flavors by country. Apple/double apple, mint, grape, watermelon, and bubblegum, ranked among the top five flavors for both genders with some variation in ranking by country. Apple/double apple was the most popular flavor for both genders in the overall sample and all countries, with the exception of females from Jordan and the UAE.

**Table 4:** Top five waterpipe flavors usually smoked by country and gender in four EMR countries, 2016 (N=2470)

*Jordan*		*OPT*		*UAE*		*Egypt*		*Total*	
*Female (%)*	*Male (%)*	*Female (%)*	*Male (%)*	*Female (%)*	*Male (%)*	*Female (%)*	*Male (%)*	*Female (%)*	*Male (%)*
Lemon mint (35.6)	Apple/double apple (58.6)	Apple/double apple (37.6)	Apple/double apple (67.8)	Grape mint (49.4)	Apple/double apple (41.2)	Apple/double apple (48.5)	Apple/double apple (46.0)	Apple/double apple (36.4)	Apple/double apple (55.2)
Apple/double apple (33.2)	Lemon mint (23.7)	Watermelon mint (35.8)	Lemon mint (25.9)	Mint (45.5)	Grape mint (40.2)	Watermelon (25.0)	Non-flavored (27.7)	Mint (16.8)	Grape (14.2)
Blueberry (25.3)	Watermelon mint (20.5)	Lemon mint (34.8)	Watermelon mint (25.4)	Apple/double apple (31.2)	Mint (27.5)	Lemon mint (13.2)	Lemon mint (23.1)	Grape (13.9)	Watermelon (11.6)
Watermelon mint (23.6)	Grape (15.9)	Mulberry (16.1)	Grape (10.3)	Grape (28.6)	Grape (24.5)	Chocolate, lemon, strawberry, non-flavored (8.8)	Grape (13.3)	Watermelon (13.4)	Mint (9.8)
Bubble gum (19.5)	Blueberry (13.3)	Cinnamon gum (15.6)	Mulberry (10.0)	Watermelon (20.8)	Watermelon (13.7)	Grape (7.4)	Watermelon (12.9)	Bubble gum (13.2)	Bubble gum (9.3)

OPT: Occupied Palestinian Territories. UAE: United Arab Emirates. Spearman correlation (r): Jordan=0.911, OPT=0.890, UAE=0.468, Egypt=0.802, Total=0.808; p<0.0001 for Jordan, OPT, Egypt and Total; p=0.05 for UAE.

### Most recent waterpipe smoking experience

Table 5 shows that the duration of smoking in the most recent smoking session varied between genders across countries and was statistically significant, except in the OPT. Almost half of the females smoked waterpipe for <0.5 hours compared to 30.5% of the males (p<0.001). Over one-third (34.1%) of males smoked ≥10 heads compared to 14.9% of the females. The difference by gender was highly statistically significant (p<0.001) overall, and in each country with the exception of the UAE. The majority of males (65.1%) and females (48.3%) in the overall sample had smoked their last waterpipe in a café/restaurant (p<0.001).

**Table 5:** Most recent waterpipe tobacco smoking session by duration of smoking, number of waterpipe heads, and place smoked in four EMR countries, 2016 (N=2470)

	*Jordan*		*OPT*		*UAE*		*Egypt*		*Total*	
	*Female n (%)*	*Male n (%)*	*Female n (%)*	*Male n (%)*	*Female n (%)*	*Male n (%)*	*Female n (%)*	*Male n (%)*	*Female n (%)*	*Male n (%)*
**Duration** (hours)										
<0.5	129 (45.6)	165 (35.1)	184 (47.9)	151 (41.0)	37 (48.1)	22 (21.8)	27 (41.5)	104 (20.4)	377 (46.6)	442 (30.5)
0.5–1.0	81 (28.6)	130 (27.7)	102 (28.6)	128 (34.8)	19 (24.7)	32 (31.7)	16 (24.6)	214 (42.0)	218 (26.9)	504 (34.8)
>1.0 and ≤2.0	49 (17.3)	128 (27.2)	66 (17.2)	63 (17.1)	16 (20.8)	34 (33.7)	9 (13.8)	103 (20.2)	140 (17.3)	328 (22.7)
>2.0	16 (5.7)	30 (6.4)	14 (3.6)	12 (3.3)	3 (3.9)	9 (8.9)	2 (3.1)	31 (6.1)	35 (4.3)	82 (5.7)
Don’t know / don’t remember	8 (2.8)	17 (3.6)	18 (4.7)	14 (3.8)	2 (2.6)	4 (4.0)	11 (16.9)	57 (11.2)	39 (4.8)	92 (6.4)
Total	283	470	384	368	77	101	65	509	827	1448
p-value*	0.012		0.160		0.006		0.001		<0.001	
**Number of waterpipe heads** (bowls)										
None	101 (36.9)	146 (31.3)	159 (42.4)	119 (32.8)	39 (51.3)	46 (47.9)	26 (44.1)	112 (23.0)	325 (41.5)	423 (30.0)
1	69 (25.2)	88 (18.9)	86 (22.9)	59 (16.3)	18 (23.7)	15 (15.6)	4 (6.8)	27 (5.6)	177 (22.6)	189 (13.4)
2–9	63 (23.0)	105 (22.5)	72 (19.2)	81 (22.3)	13 (17.1)	22 (22.9)	17 (28.8)	110 (22.6)	165 (21.0)	318 (22.5)
≥10	41 (15.0)	127 (27.3)	58 (15.5)	104 (28.7)	6 (7.9)	13 (13.5)	12 (20.3)	237 (48.8)	117 (14.9)	481 (34.1)
Total	274	466	375	363	76	96	59	486	784	1411
p-value	0.001		<0.001		0.326		<0.001		<0.001	
**Place**										
Café/restaurant	118 (41.0)	222 (46.3)	159 (41.4)	180 (51.3)	64 (83.1)	81 (84.4)	53 (80.3)	450 (88.8)	394 (48.3)	933 (65.1)
At home	88 (30.6)	132 (27.5)	132 (34.4)	76 (21.7)	8 (10.4)	8 (8.3)	1 (1.5)	11 (2.2)	229 (28.1)	227 (15.8)
Someone else’s home	64 (22.2)	98 (20.4)	64 (16.7)	54 (15.4)	3 (3.9)	4 (4.2)	5 (7.6)	21 (4.1)	136 (16.7)	177 (12.3)
University accommodation	8 (2.8)	15 (3.1)	18 (4.7)	33 (9.4)	-	-	0 (0.0)	14 (2.8)	26 (3.2)	62 (4.3)
Don’t know/don’t remember	10 (3.5)	13 (2.7)	11 (2.9)	8 (2.3)	2 (2.6)	3 (3.1)	7 (10.6)	11 (2.2)	30 (3.7)	35 (2.4)
Total	288	480	384	351	77	96	66	507	815	1434
p-value	0.659		<0.001		0.969		0.002		<0.001	

OPT: Occupied Palestinian Territories. UAE: United Arab Emirates. The numbers vary by each variable due to missing values.

* p-values for each waterpipe factor were derived from chi-squared tests.

The mean price paid for the last smoking session by females (7.58 ± 4.48 US$) across all countries was higher than that of males (4.68 ± 4.62 US$). In individual countries, females reported paying more than their male counterparts with differences that were statistically significant except for the UAE which had the highest price paid by females (14.05 ± 5.93 US$) and males (12.63 ± 8.16 US$).

## DISCUSSION

Although the gender gap in smoking prevalence is narrowing in several countries^17^, the gap is generally wide in countries of the EMR. However, recent studies indicate that the gender gap is narrowing for certain tobacco products^5^. The increasing social acceptability of female smoking in the region, the perception of reduced harm with flavored waterpipe tobacco in comparison to cigarettes and its wide accessibility are among the reasons behind the narrowing of the gap4,5,7,8.

The EMR has the highest prevalence of WTS globally, with a higher prevalence of WTS among males than females^18^. One in ten youth in the EMR smoke waterpipe, with variation between countries and a higher prevalence among males^3^. In this study, the gender gap for WTS was much lower than that of cigarette smoking. This is in accordance with previous studies from the region that reported a narrower gap for waterpipe than cigarettes^2,5,6^. As for electronic cigarettes and electronic waterpipe, the gender gap was similar. Recent evidence from Egypt indicates that ENDS use is higher among females and the gender gap in certain types of tobacco use is narrowing^5^. The gender differences with respect to age, educational level, marital status and employment might have affected the gap by making it narrower than it would have been if these differences did not exist.

Waterpipe tobacco consumption was also higher among males than females in our study. This in support to what has been reported from Jordan where the proportion of male (40.6%) dental university students, who smoked more than two sessions per week, was more than double that of females (18.6%)^19^.

Midwakh smoking was not common among the study participants except among UAE males and females. A recent study on adult UAE nationals reported that midwakh was the second most common type of smoking with a prevalence of 3.6% among males and rarely smoked by females^20^. Another UAE study on youth aged 13–15 years^21^ reported a 9.7% prevalence among males, double that of females (3.4%), while a lower prevalence (6.7% and 2.7%, respectively) was reported from Lebanon^22^ among youth aged 12–18 years. Smokeless tobacco was uncommon among waterpipe smokers in our study. This in line with what has been reported from most EMR countries, particularly among youth^23^. Smokeless tobacco is however used in Sudan and Yemen particularly among males and to a lesser extent in Egypt and Saudi Arabia^24^.

Although the majority of males and females in our study started smoking at the ages of 15–19 years, the age group is older than what has been reported for schoolchildren in a study from Jordan where 58% of males and 63% of females started to smoke between the ages of 11 and 14 years^25^. It is, however, similar to what has been reported by dental university students in Jordan^19^ where half started waterpipe smoking between 16 and 18 years.

Across all countries, the proportion of female students smoking their first waterpipe with family exceeded that of males. It is not surprising that over one-third of females started WTS with family members, which indicates the social acceptability of WTS among females in the Arab culture. The fact that half of females from the OPT smoked their first waterpipe with family members, in contrast to 1.5% of females from Egypt, shows variation in social acceptability of female WTS in countries of the region^4^. The study of dental students from Jordan found that almost half of female students smoked waterpipe in their parents’ presence in contrast to 21.7% of males^19^. WTS by females in front of their parents, relatives and in public can be attributed to the social acceptability of its use in contrast to the negative attitude towards cigarette smoking by women^4^. Studies from Iran, an EMR non-Arab country but with similar culture and traditions to Arab countries, have underscored the role of the family in WTS initiation^26^. Having higher proportions in both sexes of always smoking alone in Egypt is worrying as a recent study from Egypt reported it as an independent determinant of self-reported addiction to WTS^27^.

The fact that females paid more than males per tobacco session could be partly attributed to the increasing power of spending among women^13^ and that females do not usually go to traditional cafés that generally charge less and are mainly frequented by men.

It has been reported that women usually prefer sweet waterpipe tobacco flavors like fruits, candy and chocolate instead of other flavors and unflavored tobacco^28^. Moreover, flavored waterpipe tobacco is more popular among the young as reported from the US and the Arab region^12,15,29^. Hence, it is not surprising that only 1% of females usually smoked non-flavored tobacco in our study compared to 11% of their male counterparts. Previous studies from the region affirmed that females prefer flavored tobacco for its attractive taste and smell^30^ that motivate many to start and continue WTS^31,32^. Flavored waterpipe tobacco has abundant flavors, with ‘two apples’ being the most popular^32^. In our study, the ranks of the flavors were highly correlated between genders. Apple/double apple was the most popular flavor smoked among respondents of both sexes in the total sample, in the OPT and Egypt, and among males in Jordan and the UAE. Although not much is yet known about the potential health risks of flavored tobacco^32^, the need to have regulations to restrict flavors in waterpipe tobacco similar to cigarettes has been raised^31^.

The current study showed that the gender gap is narrowing with respect to the prevalence of WTS and age of initiation of WTS in the EMR. In addition, the socio-cultural deterrents of WTS uptake and continued smoking are diminishing for youth in both genders. Equal opportunities are given to the young in both sexes by their families and communities in our region to smoke the waterpipe. This WTS encouraging environment for females, the available varieties of flavors, the flourishing café culture and the tobacco marketing strategies that target women puts women in the EMR on equal grounds with their male peers^12,23,24,33^. Women in the EMR seem to have similar freedom as males in accessing waterpipe tobacco products and in smoking at home or outside. While Goal 5 of the Sustainable Development Goals (SDG5) calls for achieving gender equality, empowering women and abolishing discrimination against them^34^, it is not the intention of this goal to increase their access to harmful tobacco products but to provide knowledge on the WTS hazards equally to both genders^35^.

The results of our study have implications for educational establishments, tobacco control organizations and women societies to raise awareness that WTS is not a socially acceptable behavior and not to only educate them on its harms. Further, health policy makers are urged to control the expansion of the waterpipe serving establishments and ban use of flavors in tobacco products including waterpipe.

### Limitations

Our study has some limitations as it included four EMR countries and six universities, which makes it difficult to generalize to all EMR countries and university students. Further, the number of study participants varied by country and we were unable to reach the target number of students in the UAE; however, this study provides unique insights into waterpipe patterns among young adults in four EMR countries, including in the UAE. Another limitation is that the study was based on convenience sampling. However, our study is one of the few studies that have examined WTS and gender, and assessed several related factors in the region. Moreover, the use of a web survey could have increased the participation rate of females in the survey and the reliability of their answers.

## CONCLUSIONS

Male students had higher prevalence than females of all forms of tobacco smoking with a narrower gender gap for WTS. There were gender differences with respect to WTS in several aspects: age of initiation, person(s) with whom and place at which first smoked WTS, duration of smoking, tobacco consumption, place of purchase, and expenditure. The ease of accessibility of waterpipe tobacco from homes and cafés in the region make it easier for the young, and females in particular, to start smoking and maintain this habit. Further, the availability of different tobacco flavors in countries of the region calls for action as it motivates the young to start smoking, particularly females. Regular surveillance of the prevalence of WTS is needed to monitor the gender gap.
