# Supplementary material for: Gender differences in waterpipe tobacco smoking among university students in four Eastern Mediterranean countries
Source: Tob Induc Dis. 2020 Dec 2;18:100. doi: 10.18332/tid/129266 (PMC7720794; doi:10.18332/tid/129266)
Supplement: Supplementary file 1 [file TID-18-100-s1.pdf]

Table S1: Usual persons smoking waterpipe with and places of smoking and purchase in four EMR countries, 2016 (n=2470)

	Jordan		OPT		UAE		Egypt		Total	
	**Female**	**Male**	**Female**	**Male**	**Female**	**Male**	**Female**	**Male**	**Female**	**Male**
	N (%)	N (%)	N (%)	N (%)	N (%)	N (%)	N (%)	N (%)	N (%)	N (%)
	**Usual persons smoking waterpipe with**									
Alone										
Almost always/always	21 (8.3)	43 (9.4)	9 (2.9)	23 (7.7)	2 (2.8)	5 (5.3)	27 (45.0)	176 (38.3)	59 (8.5)	247 (18.9)
Usually/most of the time	16 (6.3)	44 (9.6)	7 (2.2)	23 (7.7)	3 (4.2)	7 (7.4)	7 (11.7)	66 (14.3)	33 (4.7)	140 (10.7)
Often	9 (3.6)	39 (8.6)	7 (2.2)	18 (6.0)	1 (1.4)	6 (6.3)	2 (3.3)	31 (6.7)	19 (2.7)	94 (7.2)
Sometimes	38 (15.1)	82 (18.0)	26 (8.3)	35 (11.7)	0 (0.0)	12 (12.6)	9 (15.0)	64 (13.9)	73 (10.5)	193 (14.7)
Seldom/rarely	61 (24.2)	104 (22.8)	78 (24.8)	79 (26.5)	12 (16.9)	19 (20.0)	7 (11.7)	62 (13.5)	158 (22.7)	264 (20.2)
Never	107 (42.5)	144 (31.6)	181 (57.6)	113 (37.9)	49 (69.0)	46 (48.4)	8 (13.3)	59 (12.8)	345 (49.5)	362 (27.7)
Don’t know/don’t remember	0 (0.0)	0 (0.0)	6 (1.9)	7 (2.3)	4 (5.6)	0 (0.0)	0 (0.0)	2 (0.4)	10 (1.4)	9 (0.7)
Total	252	456	314	298	71	95	60	460	696	1309
*p-value**	0.012		<0 .001		0.002		0.889		<0.001	
With one friend										
Almost always/always	59 (22.3)	77 (17.1)	41 (12.5)	50 (15.8)	15 (20.5)	13 (13.7)	26 (41.9)	191 (38.7)	141 (19.3)	331 (24.4)
Usually/most of the time	57 (21.6)	82 (18.2)	41 (12.5)	63 (19.9)	9 (12.3)	14 (14.7)	19 (30.6)	122 (24.7)	128 (17.5)	281 (20.8)
Often	36 (13.6)	93 (20.7)	52 (15.8)	58 (18.4)	6 (8.2)	24 (25.3)	4 (6.5)	87 (17.6)	98 (13.4)	262 (19.4)
Sometimes	46 (17.4)	107 (23.8)	69 (21.0)	79 (25.0)	16 (21.9)	14 (14.7)	6 (9.7)	67 (13.6)	137 (18.8)	267 (19.7)

Seldom/rarely	37 (14.0)	61 (13.6)	53 (16.1)	33 (10.4)	7 (9.6)	15 (15.8)	4 (6.5)	16 (3.2)	101 (13.8)	125 (9.2)
Never	29 (11.0)	30 (6.7)	68 (20.7)	29 (9.2)	18 (24.7)	13 (13.7)	3 (4.8)	8 (1.6)	118 (16.2)	80 (5.9)
Don't know/don't remember	0 (0.0)	0 (0.0)	5 (1.5)	4 (1.3)	2 (2.7)	2 (2.1)	0 (0.0)	2 (0.4)	7 (1.0)	8 (0.6)
Total	264	450	329	316	73	95	62	493	730	1354
p-value	0.011		<0 .001		0.040		0.107		<0.001	
With more than one friend										
Almost always/always	58 (21.6)	102 (21.7)	83 (23.8)	80 (24.2)	16 (21.3)	30 (30.0)	16 (26.7)	179 (37.1)	173 (23.0)	391 (28.3)
Usually/most of the time	59 (22.0)	124 (26.4)	64 (18.3)	89 (26.9)	19 (25.3)	14 (14.0)	14 (23.3)	138 (28.6)	156 (20.7)	365 (26.4)
Often	42 (15.7)	84 (17.9)	62 (17.8)	50 (15.1)	7 (9.3)	22 (22.0)	7 (11.7)	68 (14.1)	118 (15.7)	224 (16.2)
Sometimes	50 (18.7)	92 (19.6)	41 (11.7)	51 (15.4)	7 (9.3)	14 (14.0)	7 (11.7)	61 (12.6)	105 (14.0)	218 (15.8)
Seldom/rarely	31 (11.6)	44 (9.4)	38 (10.9)	33 (10.0)	11 (14.7)	15 (15.0)	7 (11.7)	10 (2.1)	87 (11.6)	10 2 (7.4)
Never	28 (10.4)	24 (5.1)	56 (16.0)	24 (7.3)	11 (14.7)	5 (5.0)	7 (11.7)	25 (5.2)	102 (13.6)	78 (5.6)
Don't know/don't remember	0 (0.0)	0 (0.0)	5 (1.4)	4 (1.2)	4 (5.3)	0 (0.0)	2 (3.3)	2 (0.4)	11 (1.5)	6 (0.4)
Total	268	470	349	331	75	100	60	483	752	1384
p-value	0.087		0.004		0.005		< 0.001		<0.001	
With family members										
Almost always/always	45 (17.4)	16 (3.6)	52 (15.2)	11 (3.8)	5 (6.9)	6 (6.4)	0 (0.0)	9 (2.0)	102 (14.0)	42 (3.3)
Usually/most of the time	40 (15.4)	35 (7.9)	52 (15.2)	28 (9.7)	10 (13.9)	1 (1.1)	5 (8.8)	19 (4.3)	107 (14.7)	83 (6.5)

Often	25 (9.7)	25 (5.6)	39 (11.4)	19 (6.6)	6 (8.3)	4 (4.3)	2 (3.5)	25 (5.7)	72 (9.9)	73 (5.8)
Sometimes	45 (17.4)	59 (13.3)	48 (14.1)	41 (14.2)	5 (6.9)	5 (5.3)	0 (0.0)	19 (4.3)	98 (13.4)	124 (9.8)
Seldom/rarely	36 (13.9)	94 (21.2)	65 (19.1)	68 (23.6)	14 (19.4)	17 (18.1)	6 (10.5)	56 (12.7)	121 (16.6)	235 (18.5)
Never	68 (26.3)	215 (48.4)	79 (23.2)	114 (39.6)	32 (44.4)	60 (63.8)	41 (71.9)	289 (65.4)	220 (30.2)	678 (53.5)
Don't know/don't remember	0 (0.0)	0 (0.0)	6 (1.8)	7 (2.4)	0 (0.0)	1 (1.1)	3 (5.3)	25 (5.7)	9 (1.2)	33 (2.6)
Total	259	444	40	288	72	94	57	442	729	1268
p-value	<0 .001		< 0.001		0.020		0.353		<0.001	
With others										
Almost always/always	19 (11.7)	23 (8.1)	10 (5.9)	7 (4.2)	1 (11.1)	1 (4.8)	2 (4.3)	1 (0.3)	32 (8.2)	32 (3.8)
Usually/most of the time	12 (7.4)	26 (9.1)	5 (2.9)	6 (3.6)	0 (0.0)	1 (4.8)	2 (4.3)	11 (3.0)	19 (4.9)	44 (5.2)
Often	10 (6.2)	21 (7.4)	2 (1.2)	7 (4.2)	1 (11.1)	1 (4.8)	4 (8.5)	19 (5.2)	17 (4.4)	48 (5.7)
Sometimes	8 (4.9)	29 (10.2)	7 (4.1)	11 (6.6)	1 (11.1)	4 (19.0)	3 (6.4)	8 (2.2)	19 (4.9)	52 (6.2)
Seldom/rarely	12 (7.4)	25 (8.8)	3 (1.8)	2 (1.2)	0 (0.0)	1 (4.8)	1 (2.1)	3 (0.8)	16 (4.1)	31 (3.7)
Never	101 (62.3)	161 (56.5)	91 (53.5)	67 (40.1)	4 (44.4)	9 (42.9)	28 (59.6)	178 (48.5)	224 (57.7)	415 (49.4)
Don't know/don't remember	0 (0.0)	0 (0.0)	52 (30.6)	67 (40.1)	2 (22.2)	4 (19.0)	7 (14.9)	147 (40.1)	61 (15.7)	218 (26.0)
Total	162	285	170	167	9	21	47	367	388	840
p-value	0.278		0.125		0.929		0.001		<0.001	

	Usual place for smoking waterpipe									
In a cafe/restaurant										
Almost always/always	62 (23.5)	105 (22.3)	85 (24.6)	90 (27.9)	32 (42.1)	44 (44.0)	48 (76.2)	387 (77.7)	227 (30.3)	626 (45.0)
Usually/most of the time	67 (25. 4)	107 (22.7)	69 (19.9)	71 (22.0)	16 (21.1)	21 (21.0)	11 (17.5)	76 (15.3)	163 (21.8)	275 (19.8)
Often	29 (11.0)	84 (17.8)	31 (9.0)	50 (15.5)	3 (3.9)	14 (14.0)	1 (1.6)	18 (3.6)	64 (8.5)	166 (11.9)
Sometimes	40 (15.2)	89 (18.9)	63 (18.2)	61 (18.9)	6 (7.9)	5 (5.0)	3 (4.8)	6 (1.2)	112 (15.0)	161 (11.6)
Seldom/rarely	30 (11.4)	55 (11.7)	38 (11.0)	22 (6.8)	14 (18.4)	9 (9.0)	0 (0.0)	4 (0.8)	82 (10.9)	90 (6.5)
Never	34 (12.9)	23 (4.9)	55 (15.9)	27 (8.4)	4 (5.3)	7 (7.0)	0 (0.0)	5 (1.0)	93 (12.4)	62 (4.5)
Don't know/don't remember	2 (0.8)	8 (1.7)	5 (1.4)	2 (0.6)	1 (1.3)	0 (0.0)	0 (0.0)	2 (0.4)	8 (1.1)	12 (0.9)
Total	264	471	346	323	76	100	63	498	749	1392
p-value	0.001		0.004		0.143		0.352		<0.001	
In own home										
Almost always/always	42 (16.2)	61 (13.5)	66 (19.7)	45 (15.4)	2 (2.9)	3 (3.2)	2 (3.5)	20 (4.5)	112 (15.5)	129 (10.1)
Usually/most of the time	41 (15.8)	54 (12.0)	49 (14.6)	36 (12.3)	4 (5.7)	3 (3.2)	4 (7.0)	30 (6.8)	98 (13.6)	123 (9.6)
Often	28 (10.8)	39 (8.6)	32 (9.6)	26 (8.9)	3 (4.3)	5 (5.4)	2 (3.5)	23 (5.2)	65 (9.0)	93 (7.3)
Sometimes	38 (14.6)	70 (15.5)	36 (10.7)	41 (14.0)	5 (7.1)	9 (9.7)	4 (7.0)	42 (9.5)	83 (11.5)	162 (12.7)
Seldom/rarely	37 (14.2)	69 (15.3)	59 (17.6)	55 (18.8)	7 (10.0)	10 (10.8)	6 (10.5)	47 (10.6)	109 (15.1)	181 (14.2)
Never	67 (25.8)	136 (30.2)	90 (26.9)	87 (29.8)	49 (70.0)	61 (65.6)	37 (64.9)	277 (62.7)	243 (33.7)	561 (43.9)

Don't know/don't remember	7 (2.7)	22 (4.9)	3 (0.9)	2 (0.7)	0 (0.0)	2 (2.2)	2 (3.5)	3 (0.7)	12 (1.7)	29 (2.3)
Total	260	451	335	292	70	93	57	442	722	1278
p-value	0.358		0.627		0.854		0.566		<0.001	
In someone else's home										
Almost always/always	28 (10.8)	28 (6.3)	25 (7.9)	22 (7.6)	3 (4.2)	3 (3.2)	1 (1.8)	31 (6.8)	57 (8.1)	84 (6.5)
Usually/most of the time	44 (16.9)	53 (11.9)	31 (9.8)	31 (10.7)	6 (8.5)	5 (5.3)	3 (5.4)	33 (7.2)	84 (11.9)	122 (9.5)
Often	37 (14.2)	64 (14.4)	39 (12.3)	40 (13.8)	4 (5.6)	11 (11.7)	9 (16.1)	37 (8.1)	89 (12.7)	152 (11.8)
Sometimes	64 (24.6)	139 (31.3)	83 (26.3)	79 (27.2)	10 (14.1)	12 (12.8)	4 (7.1)	63 (13.7)	161 (22.9)	293 (22.8)
Seldom/rarely	39 (15.0)	97 (21.8)	63 (19.9)	66 (22.8)	12 (16.9)	25 (26.6)	8 (14.3)	71 (15.5)	122 (17.4)	259 (20.1)
Never	42 (16.2)	52 (11.7)	71 (22.5)	49 (16.9)	35 (49.3)	37 (39.4)	27 (48.2)	210 (45.8)	175 (24.9)	348 (27.0)
Don't know/don't remember	6 (2.3)	11 (2.5)	4 (1.3)	3 (1.0)	1 (1.4)	1 (1.1)	4 (7.1)	14 (3.1)	15 (2.1)	29 (2.3)
Total	260	444	316	290	71	94	56	459	703	1287
p-value	0.014		0.752		0.537		0.126		0.302	
University accommodation										
Almost always/always	13 (5.3)	22 (5.2)	9 (3.2)	30 (10.6)	1 (1.4)	3 (3.3)	2 (3.5)	15 (3.3)	25 (3.8)	70 (5.6)
Usually/most of the time	7 (2.9)	19 (4.5)	14 (4.9)	22 (7.8)	2 (2.9)	2 (2.2)	3 (5.3)	14 (3.1)	26 (4.0)	57 (4.6)
Often	7 (2.9)	17 (4.0)	7 (2.5)	14 (4.9)	1 (1.4)	5 (5.4)	3 (5.3)	20 (4.5)	18 (2.7)	56 (4.5)
Sometimes	19 (7.8)	23 (5.5)	14 (4.9)	24 (8.5)	1 (1.4)	3 (3.3)	3 (5.3)	47 (10.5)	37 (5.6)	97 (7.8)

Seldom/rarely	15 (6.1)	28 (6.6)	15 (5.3)	25 (8.8)	2 (2.9)	2 (2.2)	5 (8.8)	30 (6.7)	37 (5.6)	85 (6.8)
Never	169 (69.3)	272 (64.5)	209 (73.3)	144 (50.9)	61 (87.1)	75 (81.5)	38 (66.7)	292 (65.0)	477 (72.7)	783 (62.8)
Don't know/don't remember	14 (5.7)	41 (9.7)	17 (6.0)	24 (8.5)	2 (2.9)	2 (2.2)	3 (5.3)	31 (6.9)	36 (5.5)	98 (7.9)
Total	244	422	285	283	70	92	57	449	656	1246
p-value	0.366		<0 .001		0.786		0.846		0.003	
In other place										
Almost always/always	8 (4.3)	13 (4.0)	4 (2.2)	6 (3.9)	0 (0.0)	1 (1.5)	0 (0.0)	2 (0.5)	12 (2.6)	22 (2.4)
Usually/most of the time	4 (2.2)	12 (3.7)	1 (0.6)	5 (3.3)	2 (4.5)	0 (0.0)	4 (8.7)	15 (4.1)	11 (2.4)	32 (3.5)
Often	9 (4.9)	24 (7.5)	0 (0.0)	1 (0.7)	0 (0.0)	6 (9.1)	5 (10.9)	16 (4.4)	14 (3.1)	47 (5.2)
Sometimes	9 (4.9)	24 (7.5)	3 (1.7)	5 (3.3)	2 (4.5)	2 (3.0)	1 (2.2)	9 (2.5)	15 (3.3)	40 (4.4)
Seldom/rarely	5 (2.7)	22 (6.9)	7 (3.9)	7 (4.6)	1 (2.3)	5 (7.6)	1 (2.2)	7 (1.9)	14 (3.1)	41 (4.5)
Never	105 (56.8)	137 (42.7)	112 (62.9)	78 (51.0)	30 (68.2)	39 (59.1)	25 (54.3)	159 (43.6)	272 (60.0)	413 (45.6)
Don't know/don't remember	45 (24.3)	89 (27.7)	51 (28.7)	51 (33.3)	9 (20.5)	13 (19.7)	10 (21.7)	157 (43.0)	115 (25.4)	310 (34.3)
Total	185	321	178	153	44	66	46	365	453	905
p-value	0.051		0.184		0.145		0.085		<0.001	

	**Place usually purchase own waterpipe products**									
Supermarket	52 (18.0)	73 (14.9)	53 (14.0)	75 (21.1)	6 (8.0)	9 (9.1)	8 (11.9)	61 (11.8)	119 (14.7)	218 (14.9)
Convenience store/mini market/produce market	14 (4.8)	24 (4.9)	22 (5.8)	33 (9.3)	2 (2.7)	2 (2.0)	2 (3.0)	25 (4.8)	40 (4.9)	84 (5.7)
Street vendor	4 (1.4)	5 (1.0)	0 (0.0)	4 (1.1)	1 (1.3)	0 (0.0)	3 (4.5)	15 (2.9)	8 (1.0)	24 (1.6)
Smoke shop or tobacco specialty store	91 (31.5)	236 (48.1)	69 (18.2)	68 (19.1)	9 (12.0)	34 (34.3)	3 (4.5)	53 (10.2)	172 (21.2)	391 (26.7)
Café/Restaurant	24 (8.3)	35 (7.1)	28 (7.4)	28 (7.9)	9 (12.0)	15 (15.2)	38 (56.7)	233 (45.0)	99 (12.2)	311 (21.2)
Friend or relative	7 (2.4)	3 (0.6)	7 (1.8)	4 (1.1)	2 (2.7)	1 (1.0)	0 (0.0)	8 (1.5)	16 (2.0)	16 (1.1)
Internet	4 (1.4)	4 (0.8)	0 (0.0)	1 (0.3)	0 (0.0)	0 (0.0)	0 (0.0)	1 (0.2)	4 (0.5)	6 (0.4)
I never buy shisha products	93 (32.2)	111 (22.6)	163 (43.0)	106 (29.8)	46 (61.3)	38 (38.4)	13 (19.4)	122 (23.6)	315 (38.9)	377 (25.8)
Abroad/ duty free	0 (0.0)	0 (0.0)	37 (9.8)	37 (10.4)	0 (0.0)	0 (0.0)	0 (0.0)	0 (0.0)	37 (4.6)	37 (2.5)
Total	289	491	379	356	75	99	67	518	810	1464
p-value	0.001		0.003		0.015		0.500		<0 .001	

OPT: Occupied Palestinian Territories. UAE: United Arab Emirates. The numbers vary by each variable due to missing values. *p-values for all characteristics were derived from chi-squared tests.

© 2020 Hamadeh R.R. et al.
